# PTEN Loss in Triple‐Negative Breast Cancer: Integrative Molecular and Clinicopathological Insights

**DOI:** 10.1155/ijbc/7879645

**Published:** 2026-08-02

**Authors:** Sandeep Kumar, Tamanna Thakur, Anjali Chadda, Santhosh Irrinki, Siddhant Khare, Amanjit Bal

**Affiliations:** ^1^ Department of Histopathology, Postgraduate Institute of Medical Education and Research (PGIMER), Chandigarh, India, pgimer.edu.in; ^2^ Department of General Surgery, Postgraduate Institute of Medical Education and Research (PGIMER), Chandigarh, India, pgimer.edu.in

**Keywords:** mutations, protein expression, PTEN, TNBC

## Abstract

**Background:**

Phosphatase and tensin homolog (PTEN) is a key tumor suppressor gene that negatively regulates the PI3K/AKT pathway. PTEN deficiency is the most frequent molecular alteration in triple‐negative breast cancer (TNBC); however, the relationship between PTEN protein loss and underlying genomic alterations remains incompletely understood.

**Methods:**

Fifty TNBC cases were evaluated for PTEN protein expression by immunohistochemistry on tissue microarrays. PTEN hotspot mutations (Exons 1, 5, 7, and 9) were analyzed using Sanger sequencing, whereas copy number alterations were assessed by multiplex ligation‐dependent probe amplification (MLPA) assay. PTEN alterations were correlated with clinicopathological characteristics. External validation of PTEN mRNA and protein expression and prognostic significance was performed using publicly available TCGA/CPTAC datasets and Kaplan–Meier plotter.

**Results:**

PTEN protein loss was observed in 41/50 TNBC cases (82%), indicating that PTEN deficiency is a frequent event in this subtype. PTEN mutations were detected in six cases (12%) and were confined exclusively to Exon 5, comprising one missense mutation (c.277C>A) and two truncating mutations (c.430del and c.433del). MLPA identified PTEN copy number alterations in four cases (8%), including heterozygous deletions and duplications. No significant association was observed between PTEN protein expression and genomic alterations, highlighting a substantial genotype–phenotype discordance. Analysis of independent public datasets confirmed significantly reduced PTEN mRNA and protein expression in TNBC compared with luminal and HER2‐positive breast cancers. Patients with low PTEN expression showed a trend toward shorter overall survival (23.5 vs. 38.9 months), though the difference did not reach statistical significance.

**Conclusions:**

PTEN protein loss is highly prevalent in TNBC but is only partially explained by mutations and copy number alterations, suggesting that additional regulatory mechanisms, such as epigenetic or posttranscriptional events, contribute to PTEN inactivation. These findings underscore the biological importance of PTEN deficiency in TNBC and support further investigation of PTEN as a potential prognostic biomarker and therapeutic target.

## 1. Introduction

Triple‐negative breast cancer (TNBC) is characterized by high tumor grade, large tumor size, and a high risk of relapse and metastasis [1, 2]. It lacks expression of the estrogen receptor (ER), progesterone receptor (PR), and human epidermal growth factor Receptor 2 (HER2) and accounts for 15%–20% of all breast cancers [3, 4]. The prevalence of TNBC among Indian women is around 27.9%, which is high compared with other regions [5]. Due to the absence of all three receptors, targeted therapies for TNBC are limited. Most patients are treated with conventional chemotherapy; however, not all respond effectively, leading to early relapse and distant metastasis.

Oncogene activation and loss of tumor suppressor gene function contribute to breast cancer invasion and metastasis [6, 7]. The phosphatase and tensin homolog (PTEN) functions as a critical tumor suppressor, and its mutation or loss of expression is prevalent across diverse human malignancies [8]. PTEN is involved in various cellular events, including cell adhesion, differentiation, migration, proliferation, apoptosis, and metastasis [9–12]. PTEN is a negative regulator of the phosphatidylinositol 3‐kinase (PI3K)/AKT signaling pathway, and its loss leads to increased expression of PI3K, particularly the PI3K beta isoform (PIK3CB), via the lipid kinase domain [13]. PTEN′s primary function is to dephosphorylate phosphatidylinositol 3,4,5‐trisphosphate, a key activator of PDK and AKT. Loss of PTEN expression results in upregulation of phosphatidylinositol 3,4,5‐trisphosphate, AKT, and the PI3K pathway, which are critical for cellular development and survival [14]. PTEN loss has also been associated with higher tumor grade, larger tumor size, lymph node involvement, and recurrence of TNBC tumors [15, 16].

Several studies have demonstrated that PTEN deficiency is among the most frequent molecular alterations in TNBC, with reported loss of protein expression ranging from approximately 40%–85%. However, considerable discordance exists between PTEN protein loss and detectable genomic alterations, suggesting that mechanisms beyond mutation and deletion contribute to PTEN inactivation. Previous studies have largely focused on either immunohistochemical assessment or genomic characterization alone, whereas comprehensive evaluations integrating protein expression, mutation profiling, copy number analysis, and external transcriptomic validation remain limited, particularly in Indian TNBC cohorts. Addressing this gap may improve understanding of PTEN biology and its potential utility as a prognostic and therapeutic biomarker. The present study is aimed at evaluating PTEN alterations in TNBC using molecular and protein‐based approaches and correlating these findings with clinical outcomes.

## 2. Materials and Methods

### 2.1. Patients and Samples

The present study was conducted on 50 female patients diagnosed with TNBC in the Departments of Histopathology and General Surgery, PGIMER, Chandigarh, India. Patients who had not received neoadjuvant chemotherapy or radiotherapy prior to surgery and had adequate tumor tissue available were included in the study. Histological subtype and tumor grade were evaluated according to the modified Bloom–Richardson grading system and were independently confirmed by two pathologists.

Fresh tumor tissue was collected from each patient during surgical excision and immediately stored at −80°C until further analysis. Tumor and adjacent normal tissues were distinguished based on histopathological examination. TNBC status was confirmed by immunohistochemistry (IHC), defined by the absence of ER, PR, and HER2 expression. Clinicopathological parameters were recorded for all patients, including age, tumor size, tumor type, histological grade, histological subtype, and lymph node metastasis status.

### 2.2. Construction of Tissue Microarray (TMA) and PTEN IHC

Triple‐negative breast tumor samples were added to a TMA using a TMA instrument (UNI‐TMA, UATM‐272B, Seoul, Korea). Briefly, tumor regions were identified microscopically on hematoxylin and eosin (H&E)–stained slides, and the corresponding areas were marked on the respective paraffin‐embedded donor blocks. From each case, three 2 mm cores were obtained from morphologically representative, nonnecrotic tumor regions and transferred into a paraffin acceptor block. The cores were positioned according to a preassigned coordinate system (X–Y guide), and their locations were documented in Microsoft Excel. A maximum of 60 cores were embedded in a single TMA block.

Paraffin‐embedded sections (2–4 *μ*m thickness) were prepared from the TMA blocks and utilized for immunohistochemical analysis. IHC was performed using an automated immunostaining platform (BenchMark XT, Ventana Medical Systems Inc., United States). PTEN immunostaining was performed with an anti‐PTEN antibody (Clone 138G6) at 1:100 dilution. Staining was evaluated independently by two pathologists in a blinded manner. TNBC cases with < 90% nuclear PTEN expression were considered to exhibit loss of PTEN expression [17].

### 2.3. Sanger Sequencing for PTEN Hotspot Mutations

Genomic DNA was extracted from fresh frozen TNBC tissue samples using a commercial DNA extraction kit (QIAamp DNA Mini Kit, Qiagen, Hilden, Germany). Concentration of DNA was measured using a spectrophotometer (NanoDrop 2000, Thermo Scientific, United States). Isolated DNA was subjected to PCR amplifications targeting PTEN (Exons 1, 5, 7, and 9) using the primer pairs (primer details are given in Supporting Information 1: Table S1). The amplified genomic DNA was purified using a commercial kit (QIAquick, Qiagen, United States) and analyzed by 2% agarose gel electrophoresis. Sequencing was performed using the BigDye Terminator V3.1 sequencing kit (Life Technologies) on ABI 3500 series genetic analyzers (Life Technologies). The sequences obtained from Sanger sequencing were visualized individually using FinchTV (PerkinElmer, Geospiza, United States).

### 2.4. Multiplex Ligation‐Dependent Probe Amplification (MLPA) Experiment

Multiplex MLPA analysis was performed using genomic DNA isolated from TNBC tumor tissue. MLPA was performed using the SALSA MLPA Reagent Kit and the Probemix P225‐E1 PTEN. Probemix contains probes for detecting deletions or duplications in the human PTEN gene. Information about this kit can be found on the website MRC‐Holland.com. Experiments were performed as per the manufacturer′s protocol, and analysis of MLPA data was carried out using Coffalyzer.net. The standard deviation of < 0.10 in all probes in the reference samples was considered. A dosage quotient (DQ) of a probe between 0.80 and 1.20 was considered normal, and a DQ of 0 was considered a homozygous deletion; between 0.40 and 0.65, a heterozygous deletion; between 1.30 and 1.65, a heterozygous triplication; and between 1.75 and 2.15, a heterozygous triplication/homozygous duplication.

### 2.5. PTEN Expression and Prognostic Significance Analysis Based on Public Datasets

The expression and prognostic significance of PTEN in breast cancer were analyzed using publicly available databases. The UALCAN database (https://ualcan.path.uab.edu), which uses data from The Cancer Genome Atlas (TCGA) and the Clinical Proteomic Tumor Analysis Consortium (CPTAC), was used to examine PTEN mRNA and protein expression across breast cancer subtypes [18, 19]. The prognostic relevance of PTEN mRNA expression in TNBC patients who did not receive chemotherapy was evaluated using the Kaplan–Meier plotter tool (https://kmplot.com/analysis) based on RNA‐seq datasets [20]. Patients were divided into high and low PTEN expression cohorts, and upper‐quartile survival times (in months) were reported for each group. Hazard ratios (HRs) with 95% confidence intervals (CIs) and log‐rank *p* values were calculated automatically by the platform.

### 2.6. Statistical Analysis

Correlation between PTEN expression, genomic aberrations, and various clinicopathological variables, such as age, tumor size, grade, histological subtypes, lymph node status, and Ki‐67, was assessed using bivariate analysis with the chi‐square test or Fisher′s exact test, as appropriate. *p* values of less than 0.05 were considered statistically significant.

## 3. Results

### 3.1. Characteristics of the Study Population

Fifty TNBC cases were diagnosed based on negative ER, PR, and HER2 expression. The mean age of the TNBC cohort was 50.24 years (SD 9.9, range 30–72 years). The mean tumor size was 2.8 cm (range 1–5 cm). Infiltrating duct carcinoma, no special type (*n* = 44, 88%) was the commonest histological subtype, and histological Grade III tumors constituted the majority of cases (47 cases, 94%). Lymph node metastasis was noted in 34% (17/50) of cases. Clinicopathological data of these cases are given in Table 1.

**Table 1:** Clinicopathological features of TNBC patients (*N* = 50).

Characteristics (*n* = 50)			Number (%)
Age (years)	Mean 50.24	< 30	0
	SD ±9.9	30–50	27 (54)
	Range 30–72	> 50	23 (46)

Tumor size (cm)	Mean 2.8 cm	≤ 2	18 (36)
	SD ±1.06	2–5	32 (64)
	Range 1–5 cm	> 5	0

Lymph node status	Positive		17 (34)
	Negative		33 (66)

Histological grade	II		3 (6)
	III		47 (94)

Histological subtypes	Invasive ductal carcinoma (IDC), NST		44 (88)
	Others		6 (12)

Ki‐67	Mean 50.5%	≤ 14%	1 (2)
	SD ±22.6	> 14%	49 (98)
	Range 5%–90%		

### 3.2. Immunohistochemical Expression of PTEN

**Figure 1:**
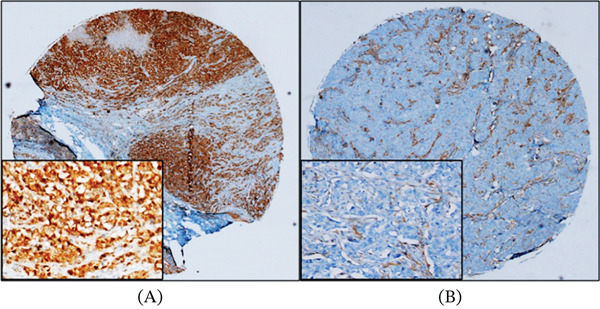
Immunohistochemical analysis of PTEN expression in triple‐negative breast cancer (TNBC) tissue samples. Representative photomicrographs of PTEN immunohistochemical staining performed on tissue microarray (TMA) sections from TNBC cases (*n* = 50). PTEN protein expression was assessed using anti‐PTEN antibody (Clone 138G6, 1:100 dilution). (A) Tumor tissue showing retained PTEN expression. (B) Tumor tissue showing loss of PTEN expression, defined as < 90% nuclear staining in tumor cells. All sections were counterstained with hematoxylin.

PTEN protein expression was evaluated because PTEN loss is one of the most frequent alterations affecting the PI3K/AKT pathway in TNBC and may occur even in the absence of detectable genomic alterations. To assess PTEN protein status across the cohort, IHC was performed on TMA sections containing triplicate cores from each tumor. Representative images of retained and lost PTEN expression are shown in Figure 1A,B, respectively. PTEN loss was defined as nuclear staining in fewer than 90% of tumor cells. Using these criteria, PTEN loss was identified in 41 of 50 cases (82%), whereas only 9 cases (18%) retained PTEN expression. The high frequency of PTEN loss observed at the protein level suggests that disruption of PTEN signaling is a common event in TNBC and supports further investigation of the underlying molecular mechanisms explored in subsequent sections.

### 3.3. Spectrum of Hotspot Mutations in the PTEN Gene

**Figure 2:**
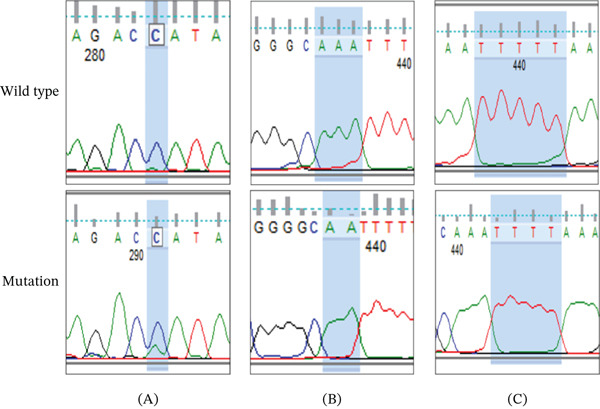
Sanger sequencing analysis of PTEN gene Exon 5 mutations in triple‐negative breast cancer (TNBC) tumor tissues. Sanger sequencing chromatograms showing wild‐type and mutant PTEN sequences in Exon 5 obtained from genomic DNA of TNBC tumor samples. The upper chromatograms represent wild‐type sequences, whereas the lower chromatograms show corresponding mutant sequences. Identified alterations include (A) a nucleotide substitution c.277C>A, (B) a single‐nucleotide deletion c.430del (deletion A), and (C) a single‐nucleotide deletion c.433del (deletion T). All detected mutations were confined to Exon 5 of the PTEN gene. Chromatograms were analyzed using FinchTV software (PerkinElmer, Geospiza, United States).

To determine whether PTEN protein loss could be explained by underlying genetic alterations, hotspot mutation analysis was performed using Sanger sequencing of PTEN Exons 1, 5, 7, and 9, which encompass commonly reported mutational hotspots. Representative chromatograms are shown in Figure 2. Pathogenic alterations were detected exclusively in Exon 5, highlighting this region as the predominant mutational hotspot in the present cohort. Three mutations were identified: c.277C>A (missense), c.430del (frameshift), and c.433del (stop–gain). Overall, PTEN mutations were detected in 6 of 50 tumors (12%) (Table 2). Despite the high prevalence of PTEN protein loss, mutation frequency remained relatively low, and no statistically significant association was observed between PTEN mutation status and PTEN immunohistochemical expression. These findings indicate that PTEN protein loss in TNBC is not solely attributable to coding sequence mutations and suggest the involvement of additional regulatory mechanisms. Clinicopathological details of PTEN IHC and PTEN mutation cases are given in Supporting Information 2: Table S2.

**Table 2:** PTEN mutations in TNBC patients (*N* = 50).

PTEN (exon)	Nucleotide change	Effect on protein	Mutation type	Previously reported	Patients	Frequency
5	c.277C>A	H [CAT] > N [AAT]	Nonsynonymous	dbSNP (rs786204927)	1	2%
				ClinVar (VCV000523356)		
5	c.430del (deletion A)	K [AAA] > N [AA]	Frameshift variant	dbSNP (rs1114167657)	3	6%
				ClinVar (VCV000428236.2)		
5	c.433del (deletion T)	L [TTA] > ∗ [TA]	Stop gained	dbSNP (rs1114167627)	2	4%
				ClinVar (VCV000428201.2)		
Total					6	12%

### 3.4. MLPA for the PTEN Gene

**Figure 3:**
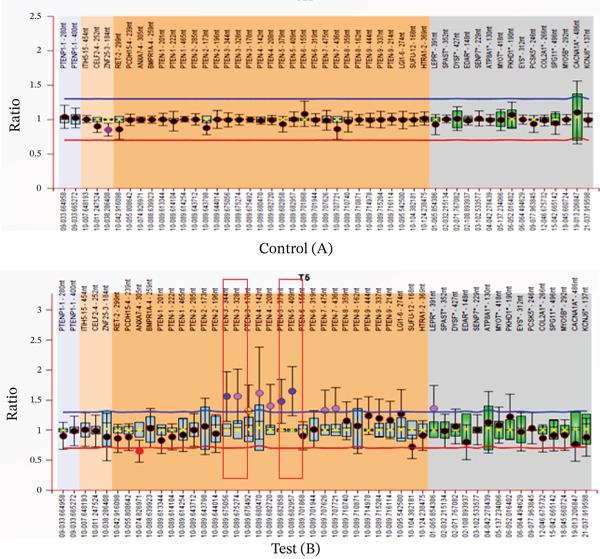
Representative MLPA analysis showing *PTEN* duplications. Multiplex ligation‐dependent probe amplification (MLPA) analysis using the SALSA P225‐E1 Probemix revealed duplications at PTEN Exons 3 and 5 (PTEN‐3dup and PTEN‐5dup probes) in (B) a TNBC test sample compared with (A) the control sample. Control samples were included for assay validation.

Because PTEN inactivation may also occur through copy number alterations, MLPA analysis was undertaken to evaluate deletions and duplications across the PTEN locus. MLPA identified copy number abnormalities in 4 of 50 tumors (8%). Representative MLPA profiles are illustrated in Figure 3. Detected alterations included heterozygous duplications involving PTEN‐3dup, PTEN‐5dup, and PTEN‐7dup probes, as well as heterozygous deletions involving PTEN‐1 and PTEN‐6 probes. All alterations were mapped to Chromosome 10q23.31, the genomic location of PTEN. Although copy number abnormalities were identified in a subset of tumors, their frequency was substantially lower than the frequency of PTEN protein loss. These findings further support the notion that genomic alterations alone do not fully account for PTEN deficiency in TNBC.

### 3.5. PTEN Expression and Prognostic Evaluation in TNBC

**Figure 4:**
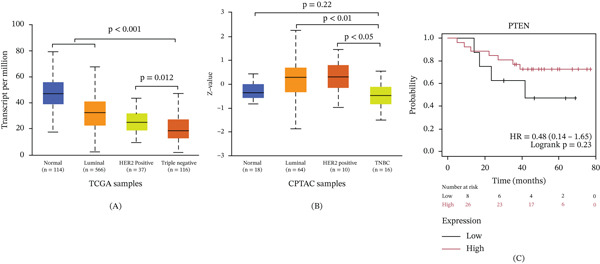
(A) Expression of PTEN in BRCA based on breast cancer subclasses. PTEN mRNA expression was significantly lower in TNBC compared with normal and Luminal A subtypes (*p* < 0.001) and also lower than in HER2‐positive tumors (*p* = 0.012). (B) PTEN protein expression showed no significant difference versus normal tissue but was markedly reduced in TNBC compared with Luminal A (*p* < 0.01) and HER2‐positive (*p* < 0.05) subtypes. Overall, both mRNA and protein levels of PTEN were lowest in TNBC. (C) The upper‐quartile overall survival in TNBC patients with low and high PTEN mRNA expression was 23.5 and 38.9 months, respectively (*p* = 0.23).

To place the findings of our cohort within a broader biological context, PTEN expression and prognostic significance were examined using TCGA and CPTAC datasets via UALCAN. Transcriptomic analysis from TCGA demonstrated significantly lower PTEN mRNA expression in TNBC compared with normal breast tissue, Luminal A tumors (*p* < 0.001), and HER2‐positive cancers (*p* = 0.012) (Figure 4A). Proteomic analysis revealed a similar pattern, with PTEN protein expression being lowest in TNBC relative to other molecular subtypes (PTEN protein levels were markedly decreased in TNBC compared with Luminal A [*p* < 0.01] and HER2‐positive [*p* < 0.05] cancers) (Figure 4B). These independent datasets, therefore, validate the predominance of PTEN deficiency observed in our cohort.

To explore the clinical relevance of PTEN expression, survival analysis was performed using the Kaplan–Meier plotter platform (Figure 4C). Patients with high PTEN expression exhibited longer upper‐quartile overall survival than those with low expression (38.9 vs. 23.5 months), although the difference did not reach statistical significance. Collectively, these data support a biologically relevant role for PTEN loss in TNBC while emphasizing the need for larger cohorts to clarify its prognostic value.

## 4. Discussion

TNBC remains one of the most clinically challenging breast cancer subtypes due to its aggressive behavior, molecular heterogeneity, and limited therapeutic options [21]. Aberrant activation of the PI3K/AKT signaling pathway is a frequent event in TNBC, and PTEN functions as a key negative regulator of this pathway. Consequently, PTEN deficiency has emerged as a biologically important event that may contribute to tumor progression, therapeutic resistance, and adverse clinical outcomes [22]. In the present study, we performed an integrated evaluation of PTEN alterations in TNBC using IHC, hotspot mutation analysis, MLPA‐based copy number analysis, and validation using publicly available transcriptomic and proteomic datasets. Our findings provide insight into the complex mechanisms underlying PTEN deficiency and highlight the substantial discordance between PTEN protein loss and detectable genomic alterations.

One of the most striking observations in our cohort was the high frequency of PTEN protein loss, identified in 82% of TNBC cases. This finding is consistent with previous studies reporting PTEN loss in approximately 69%–85% of TNBC tumors. Beg et al. [23] reported PTEN loss in 85% of Middle Eastern TNBC cases, whereas Millis et al. [24] observed PTEN deficiency in nearly 70% of TNBC tumors using large‐scale molecular profiling approaches. Collectively, these studies, together with our findings, suggest that PTEN loss represents one of the most common molecular abnormalities in TNBC. No statistically significant correlation was observed between PTEN expression and clinicopathological parameters. However, other studies have reported a stronger association between PTEN mRNA downregulation and features such as tumor size and lymph node metastasis [25, 26].

Although PTEN protein loss was highly prevalent, only 12% of tumors harbored detectable PTEN mutations. All pathogenic variants were localized to Exon 5, which encodes the catalytic phosphatase domain of PTEN and is recognized as a mutational hotspot across multiple human cancers. The predominance of Exon 5 mutations observed in our cohort is consistent with previous reports showing that a substantial proportion of PTEN mutations cluster in this region, reflecting its critical role in phosphatase activity and tumor suppressor function. Similar mutation frequencies have been reported in TNBC cohorts from both Asian and Western populations, suggesting that PTEN mutations contribute to, but do not fully account for, PTEN deficiency in breast cancer [27–32].

A notable finding of the present study was the marked discrepancy between PTEN protein loss and the relatively low frequency of genomic alterations. Whereas PTEN loss was observed in the majority of cases, mutations and copy number alterations were detected in only 12% and 8% of tumors, respectively. Furthermore, no significant association was identified between PTEN immunohistochemical status and mutation status. This genotype–phenotype discordance has been reported by several investigators and represents an important biological feature of PTEN regulation. Our findings suggest that genomic alterations alone cannot explain the widespread loss of PTEN protein expression in TNBC.

Several alternative mechanisms may contribute to PTEN inactivation. Epigenetic silencing through promoter methylation has been reported in breast cancer and may suppress PTEN transcription in the absence of genomic alterations [33, 34]. Posttranscriptional regulation by microRNAs, including miR‐21, miR‐214, and miR‐221/222, has also been shown to reduce PTEN expression and promote PI3K/AKT pathway activation. In addition, PTEN protein stability can be influenced by ubiquitination, proteasomal degradation, altered intracellular localization, and posttranslational modifications. These mechanisms provide plausible explanations for the high prevalence of PTEN protein loss observed in our cohort despite relatively infrequent mutations or copy number changes. The present findings therefore support the concept that PTEN deficiency in TNBC is a multifactorial event involving both genetic and nongenetic regulatory mechanisms.

To further explore PTEN biology in TNBC, we interrogated publicly available TCGA and CPTAC datasets. Consistent with our findings, both PTEN mRNA and protein expression were significantly lower in TNBC compared with Luminal A and HER2‐positive breast cancers [35]. These observations provide independent validation of our results and strengthen the evidence that PTEN downregulation is a characteristic feature of TNBC. Importantly, the concordance between our protein‐level observations and external transcriptomic and proteomic datasets suggests that PTEN loss is not restricted to a specific patient population but represents a broader biological phenomenon across TNBC.

The prognostic significance of PTEN remains an area of active investigation. In the present study, Kaplan–Meier analysis demonstrated a trend toward improved overall survival in patients with higher PTEN expression, although the difference did not reach statistical significance. Similar findings have been reported in previous studies, in which PTEN loss was associated with aggressive clinicopathological features but did not consistently emerge as an independent prognostic factor in multivariable analysis [36, 37]. The lack of statistical significance in our analysis may reflect limited sample size, biological heterogeneity within TNBC, and the influence of co‐occurring molecular alterations. Nevertheless, the observed survival trend supports the hypothesis that PTEN preservation may confer a more favorable biological phenotype.

The clinical implications of PTEN loss extend beyond prognostication. Increasing evidence indicates that PTEN deficiency may influence response to targeted therapies, including PI3K, AKT, and mTOR inhibitors. Furthermore, PTEN loss has been implicated in modulating the tumor immune microenvironment and may affect responsiveness to immunotherapeutic approaches. Although these therapeutic implications were not directly evaluated in the present study, our findings provide additional rationale for incorporating PTEN assessment into future biomarker‐driven clinical studies. Importantly, the discordance between PTEN genomic alterations and protein expression suggests that protein‐level evaluation may yield clinically relevant information beyond that captured by sequencing alone.

The present study contributes to the existing literature by integrating protein expression, mutation analysis, copy number evaluation, and external dataset validation within a single TNBC cohort. However, there are several limitations, including that the study was conducted at a single institution with a relatively modest sample size, which may limit generalizability. In addition, only selected hotspot exons were sequenced by Sanger, and comprehensive genomic profiling was not performed. Most importantly, functional studies investigating the biological consequences of PTEN loss were beyond the scope of this work. Future investigations incorporating methylation analysis, transcriptomic profiling, proteomic characterization, and functional validation in experimental models will be essential to define the mechanisms underlying PTEN deficiency and to determine its therapeutic relevance.

In conclusion, our findings demonstrate that PTEN loss is highly prevalent in TNBC but is only partially explained by mutations and copy number alterations. The substantial discordance between PTEN protein expression and genomic status highlights the complexity of PTEN regulation and suggests the involvement of multiple complementary mechanisms. These observations reinforce the importance of PTEN as a key molecular determinant of TNBC biology and support further investigation of PTEN‐directed biomarker and therapeutic strategies.

## Author Contributions

**Sandeep Kumar**: conceptualization, methodology, validation, investigation, and writing original draft; **Dr. Amanjit Bal**: concept design, data interpretation, funding acquisition, validation, and manuscript revision; **Tamanna Thakur**: formal analysis, data interpretation, and manuscript revision; **Anjali Chadda**: data interpretation and visualization; **Dr. Santhosh Irrinki** and **Dr. Siddhant Khare**: clinical information and manuscript revision.

## Funding

No funding was received for this manuscript.

## Disclosure

All authors declare their consent to the publication of this work.

## Conflicts of Interest

The authors declare no conflicts of interest.

## Supporting Information

Additional supporting information can be found online in the Supporting Information section.

## Supporting information


**Supporting Information 1** S1: Lists of the PTEN gene primer sequences (Exons 1, 5, 7, and 9) used for PCR amplification and subsequent Sanger sequencing analyses, including the forward and reverse primer sequences and the expected product sizes for each targeted PTEN region.

**Supporting Information 2** Table S2: The correlation between PTEN immunohistochemistry (IHC) expression levels and PTEN mutation status in a cohort of 50 triple‐negative breast cancer (TNBC) patients. The cohort is further stratified into IHC‐positive versus IHC‐negative and mutation‐positive versus mutation‐negative groups, summarizing both molecular and protein‐level findings.

## Data Availability

The datasets are available from the corresponding author on reasonable request.
