# Supplementary material for: PTEN Loss in Triple‐Negative Breast Cancer: Integrative Molecular and Clinicopathological Insights
Source: Int J Breast Cancer. 2026 Aug 2;2026:7879645. doi: 10.1155/ijbc/7879645 (PMC13430043; doi:10.1155/ijbc/7879645)
Supplement: Supplementary file 1 — Supporting Information 1 S1: Lists of the PTEN gene primer sequences (Exons 1, 5, 7, and 9) used for PCR amplification and subsequent Sanger sequencing analyses, including the forward and reverse primer sequences and the expected product sizes for each targeted PTEN region. [file IJBC-2026-7879645-s002.docx]

Tab.S1: PTEN primers sequences used for PCR amplifications and direct sequencing.

| Gene  PTEN | Sequence (5’-3’) | Annealing Temp(°C) | Size (bp) |
| --- | --- | --- | --- |
| Exon1 | Forward AGTCCAGAGCCATTTCCATCCT  Reverse GCATCCGTCTACTCCCACGTTC | 60 | 244 |
| Exon5 | Forward GACCTATGCTACCAGTCCGTAT  Reverse TTCTCAGATCCAGGAAGAGGAAAGG | 60 | 574 |
| Exon7 | Forward ACCATGCAGATCCTCAGTTTGT  Reverse TCTCACCAATGCCAGAGTAAGCAAA | 61.9 | 317 |
| Exon9 | Forward AGATGAGTCATATTTGTGGGTTTTC  Reverse ACAAGTGTCAAAACCCTGTGG | 60 | 411 |
