# Supplementary material for: PTEN Loss in Triple‐Negative Breast Cancer: Integrative Molecular and Clinicopathological Insights
Source: Int J Breast Cancer. 2026 Aug 2;2026:7879645. doi: 10.1155/ijbc/7879645 (PMC13430043; doi:10.1155/ijbc/7879645)
Supplement: Supplementary file 2 — Supporting Information 2 Table S2: The correlation between PTEN immunohistochemistry (IHC) expression levels and PTEN mutation status in a cohort of 50 triple‐negative breast cancer (TNBC) patients. The cohort is further stratified into IHC‐positive versus IHC‐negative and mutation‐positive versus mutation‐negative groups, summarizing both molecular and protein‐level findings. [file IJBC-2026-7879645-s001.docx]

Tab.S2: PTEN IHC expression and PTEN mutation correlation in TNBC patients, N=50

| Variable | Total Cohort  (N = 50) | PTEN IHC Negative  (N =41) | PTEN IHC Positive  (N=9) | P- value | PTEN Mutation negative (N=44) | PTEN Mutation positive  (n = 6) | P-value |
| --- | --- | --- | --- | --- | --- | --- | --- |
| Age, median  (range) | 50  (30 – 72) | 50  (32-72) | 50  (30-68) | 0.085 | 50  (30-72) | 52  (45-65) | 0.288 |
| Tumor Size, cm, median (range) | 3  (1-6) | 2.5  (1-6) | 3  (1.5-4) | 0.046 | 3  (1-6) | 2.6  (1.5-4) | 0.547 |
| Histological Grade, N (%)  Grade II  Grade III | 3 (6)  47 (94) | 4 (9.8)  37 (90.2) | 1 (11.1%)  8 (88.9) | 0.646 | 4 (9.1)  40 90.9) | 1 (16.7)  5 (83.3) | 0.487 |
| Lymph Node, N (%)  Positive  Negative | 17 (34)  33(66) | 17 (41.5)  24 (58.5) | 2 (22.2)  7 (77.8) | 0.247 | 18 (40.9)  26 (59.1) | 1 (16.7)  5 (83.3) | 0.249 |
| Histological subtypes, N (%)  IDC, NST  Others | 44 (88)  6 (12) | 37 (90.2)  4 (9.8) | 7 (77.8)  2 (22.2) | 0.293 | 39 (88.6)  5 (11.4) | 5 (83.3)  1 (16.7) | 0.556 |
| Ki67, %age, median (range) | 50  (5-90) | 50  (5-90) | 60  (65-90) | 0.722 | 55  (5-90) | 50  (40-90) | 0.144 |
